# Willingness to pay for social health insurance among informal sector workers in Wuhan, China: a contingent valuation study

**DOI:** 10.1186/1472-6963-7-114

**Published:** 2007-07-20

**Authors:** Till Bärnighausen, Yuanli Liu, Xinping Zhang, Rainer Sauerborn

**Affiliations:** 1 Africa Centre for Health & Population Studies, Mtubatuba, University of KwaZulu-Natal, South Africa; 2 Harvard School of Public Health, Department of Population and International Health, Boston, USA; 3 Centre for Health Care Administration, Tongji Medical College, Huazhong University of Technology and Science, Wuhan, China; 4 University of Heidelberg, Department of Tropical Hygiene and Public Health, Heidelberg, Germany

## Abstract

**Background:**

Most of the about 140 million informal sector workers in urban China do not have health insurance. A 1998 central government policy leaves it to the discretion of municipal governments to offer informal sector workers in cities voluntary participation in a social health insurance for formal sector workers, the so-called 'basic health insurance' (BHI).

**Methods:**

We used the contingent valuation method to assess the maximum willingness to pay (WTP) for BHI among informal sector workers, including unregistered rural-to-urban migrants, in Wuhan City, China. We selected respondents in a two-stage self-weighted cluster sampling scheme.

**Results:**

On average, informal sector workers were willing to pay substantial amounts for BHI (30 Renminbi (RMB), 95% confidence interval (CI) 27-33) as well as substantial proportions of their incomes (4.6%, 95% CI 4.1-5.1%). Average WTP increased significantly when any one of the copayments of the BHI was removed in the valuation: to 51 RMB (95% CI 46-56) without reimbursement ceiling; to 43 RMB (95% CI 37-49) without deductible; and to 47 RMB (95% CI 40-54) without coinsurance. WTP was higher than estimates of the cost of BHI based on past health expenditure or on premium contributions of formal sector workers. Predicted coverage with BHI declined steeply with the premium contribution at low contribution levels.

When we applied equity weighting in the aggregation of individual WTP values in order to adjust for inequity in the distribution of income, mean WTP for BHI increased with inequality aversion over a plausible range of the aversion parameter. Holding other factors constant in multiple regression analysis, for a 1% increase in income WTP for BHI with different copayments increased by 0.434-0.499% (all p < 0.0001), and for a 1% increase in past health care expenditure WTP increased by 0.076-0.148% (all p < 0.0004).  Being male, a migrant, or without permanent employment significantly decreased WTP for BHI. Education was not a significant determinant of WTP for BHI.

**Conclusion:**

Our results suggest that Chinese municipal governments should allow informal sector workers to participate in the BHI. From a normative perspective, BHI for informal sector workers is likely to increase social welfare because average WTP for BHI is significantly higher than estimates of the average cost of BHI. We further find that informal sector workers do not value the BHI as a mechanism to recover the relatively frequent but small financial losses associated with common illnesses, but because it protects against the rare but large financial losses associated with catastrophic care. From a behavioural perspective, our results predict that at a price equal to the average premium contribution of formal sector workers 35% of informal sector workers will enrol in the BHI. Subsidies and changes in insurance attributes (e.g. including catastrophic care and portability) should be effective in increasing BHI coverage. In addition, coverage should expand with rising incomes among informal sector workers in China. Finally, adverse selection will be unlikely to be a large problem, if the BHI is offered to informal sector workers.

## Background

Health insurance coverage in China's cities is linked to employment. Most formal sector employees are currently covered by one of three social health insurance systems. The Labour Insurance System (LIS, *laobao yiliao*) and the Government Insurance System (GIS, *gongfei yiliao*) were established in 1951 and 1952, respectively [1]. The LIS covers workers in large state-owned enterprises (SOEs) and collective-owned enterprises (COEs); the GIS covers current and retired government staff as well as college and university students [2-4]. In 1998, a third social health insurance for urban formal sector workers, the so-called Basic Health Insurance (BHI, *jiben yiliao baoxian*), was created by a central government decision. The BHI covers all those who are eligible for LIS or GIS and, additionally, formal workers in private sector companies and smaller public enterprises [5-8]. The BHI has replaced the LIS and GIS in a number of Chinese cities and should, according to the 1998 decision, completely replace the LIS and GIS in all Chinese cities over time. It has been estimated that in 2003, 27% [9,10] to 30% [11] of China's 500 million urban residents had BHI coverage and 25% [11] to 27% [9] had other health insurance coverage (including LIS, GIS and commercial health insurance), while between 45% [11] and 46% [9] did not have any health insurance.

**Figure 1:**
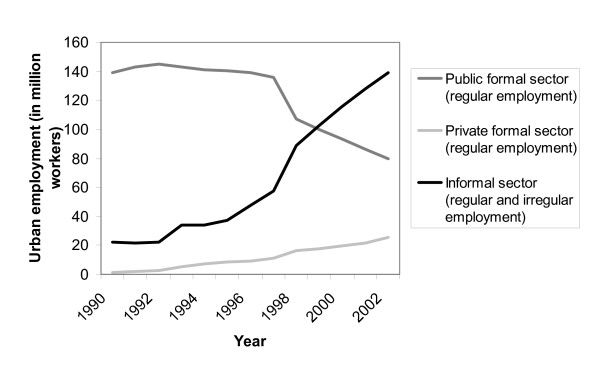
Structure of urban employment in China, 1990–2002.

In contrast to formal sector workers, most informal sector workers in Chinese cities do not have health insurance. Until the end of the Cultural Revolution in 1976 almost all workers in urban China were employed in the formal sector. Since the Chinese market reforms in the 1980s, however, the number of informal sector workers has risen dramatically as public sector enterprises laid off large numbers of employees and rural-to-urban migration increased. Most laid-off workers and rural-to-urban migrants have found (re-)employment in the informal rather than in the formal sector and are thus not eligible for health insurance coverage either in the GIS, LIS or BHI. Figure 1 shows the changes in employment structure in urban China from 1990 to 2002 based on Ghose (2005) [12]. By 2002, 57% of all urban workers (or 139 million people) were employed in the informal sector. The growth of the informal sector workforce in the past ten years is not unique to China. In many other Asian countries, similar trends have been observed [13].

Lack of health insurance coverage in China impedes access to health care [14]. Chinese health statistics show that 20% of urban residents forgo hospital inpatient care recommended by a health care professional because they are unable to pay for it [15]. Lack of health insurance is also a major cause of poverty in China [16]. A recent survey found that more than 32 million people in China (or 3% of the population) live in poverty (defined as living on less than $1.08 per day at 1993 purchasing power parity) because of out-of-pocket spending for health care [17].

One option to try to ensure that informal sector workers in urban China have access to health care and are protected against the financial risk of health expenditure is to include them in the BHI. The 1998 central government policy on BHI leaves it to the discretion of municipal governments to offer voluntary participation in the BHI to informal sectors workers [5,7,18]. To provide urban informal workers, especially rural-to-urban migrants, access to basic health care is one of China's current development priorities [19,20].

We used the contingent valuation method to assess the maximum willingness to pay (WTP) for BHI among informal sector workers in Wuhan City, China. The BHI as stipulated by the central government decision covers a comprehensive set of in- and outpatient health services with a reimbursement ceiling (i.e. the maximum Renminbi (RMB) amount that the BHI will pay per insured worker in any given year), a deductible (i.e. a fixed RMB amount that the insured person has to pay out-of-pocket in any given year before the BHI starts to make payments for covered benefits), and coinsurance (i.e. the percentage of health expenditure that the insured person has to pay out-of-pocket after the deductible has been paid) [8]. In addition to valuing the BHI as it has been laid out by the central government (henceforth called baseline BHI), we asked respondents to value separately three variations of the BHI (BHI without ceiling, BHI without deductible, and BHI without coinsurance).

Information about WTP for BHI can be used from a normative perspective and a behavioural perspective [21]. The maximum WTP equals the compensating gain, i.e. the income reduction that would maintain a respondent's initial level of utility, if he or she were provided BHI coverage [22-24]. From a normative perspective, it can thus be used as a direct measure of the monetary value of the BHI, for instance, in a cost-benefit analysis to inform policy makers about whether it will be socially desirable to provide the BHI to informal sector workers. From a behavioural perspective, demand curves constructed from the WTP results can inform policy makers about the trade-off between insurance coverage and cost recovery if the BHI were offered to informal sector workers in the market place. Our results may further aid in deciding on insurance attributes and designing marketing strategies for the BHI for informal sector workers.

## Methods

### Contingent valuation method

Contingent valuation is a survey method to elicit the maximum WTP for a good. First, the good and a hypothetical market in which the good can be bought are described to the respondent (the contingency). The respondent is then asked to state the maximum amount s/he would be willing to pay for the good (the valuation). A number of previous studies have used contingent valuation to measure the WTP for health insurance in developing countries, including in rural Burkina Faso [25-28], rural Cameroon [29,30], rural India [31], rural Iran [32], rural Nigeria [33], and Ghana [34]. A recent study has assessed the WTP for private health insurance in four small cities in China (in Shandong Province and Sichuan Province) [35]. This study differs from ours in four important aspects. First, its target population contains all types of registered urban workers, including public and private formal sector workers, while we focus exclusively on informal sector workers. Second, it investigates the WTP for different non-comprehensive private health insurances (insurance for catastrophic diseases vs. inpatient care vs. outpatient care) without copayments, while our study measures the WTP to participate in a comprehensive social health insurance with different copayments. Third, 40% of the sample in the study had public health insurance (i.e. the insurance to be valued is supplementary to public health insurance), while people with health insurance were not eligible to participate in our study. Fourth, the study took place in four small cities (one with a population of 110,000 and three with populations 30,000 – 60,000), while our study took place in one of China's ten largest cities (with a population of 3.9 million in 1999). Chinese small cities differ significantly from large cities. They are much less urbanized and economically developed than large cities [36] and have lower insurance coverage [37].

### Choice of WTP question format

We chose the payment card format to elicit WTP values. Respondents were handed a card with a number of monetary values in ascending order and were asked to circle all those monetary values they would be willing to pay for the BHI up to and including the maximum amount. The payment card format was originally developed by Mitchell and Carson [22] and has been used in a number of previous studies evaluating WTP for health care [38]. It has a number of advantages over alternative question formats. Unlike the 'take-it-or-leave it' (TIOLI) format and the bidding game format it does not suffer from starting point bias and unlike the TIOLI format it does not suffer from yeah-saying bias [39]. Theoretically, payment card questions are vulnerable to forms of range bias (i.e. respondents' answers are influenced by the highest monetary value on the card) and mid-point bias (i.e. respondents tend to state their maximum WTP in the middle of the card). However, there is little empirical evidence of the existence of range bias or mid-point bias [40,41]. In addition, while the payment card method could give rise to 'protest zeros' (i.e. false zero valuations in order to express dissent to some attribute of the WTP question), unlike the open-ended WTP question format it has not been found to lead to a very high proportion of protest-zero responses [40-43]. In order to distinguish between protest zeros and true zeros in this survey, all respondents who did not give at least one non-zero response to one of the four WTP questions were asked for the reason for their unwillingness to pay using a multiple choice question with an open-ended category.

### Setting and sample

The survey was conducted between September 1999 and January 2000 in Wuhan City, Hubei Province, China. Wuhan is a major port and industrial city in central China located alongside the Yangtze River. We chose Wuhan as the site for this study, because it is typical of the average Chinese city with regard to age composition, educational attainment and income [44]. The survey was conducted by fifteen interviewers who at the time were either graduate or postgraduate students in Public Health at the National Centre for Health Management at Tongji Medical University in Wuhan, China. All of the interviewers had previously taken part in population-based surveys and were specifically trained for the WTP survey. The study was reviewed and approved by the medical ethics committee of Tongji Medical University. Before the main survey was conducted, 65 respondents were interviewed in a pilot survey. As a result of the experiences in the pilot study, some survey questions were changed.

The target population were informal sector workers in Wuhan who were at least 18 years of age but not older than 60 years of age and did not currently have any type of health insurance. We used an operational definition of informal sector workers that is similar to one that has been used in surveys conducted by the *International Labour Office*, namely own-account workers (excluding administrative workers and professionals), unpaid family workers, and employers and employees working in establishments with less than 10 persons engaged [45].

**Figure 2:**
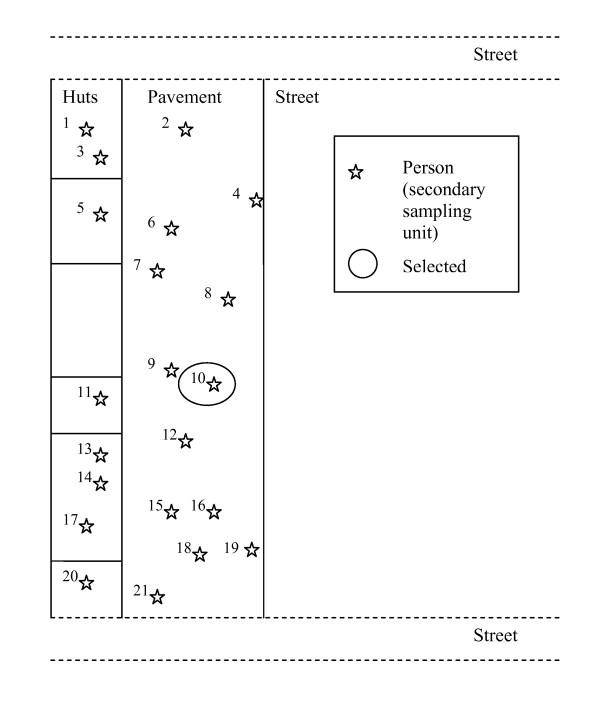
Primary sampling unit (illustrative).

A sampling frame of all informal sector workers in Wuhan did not exist because many of the rural-to-urban migrants in Wuhan are not officially registered. We defined migrants as survey participants who stated that their household registration (*hukou*) was not in Wuhan City. In order to select a representative sample of informal sector workers, we chose a two-stage cluster sampling scheme. Our primary sampling units were sides of city blocks during one of two six hour time intervals (8:00 am to 2:00 pm and 2:00 pm to 8:00 pm), i.e. there were four primary sampling units per city block. In the first sampling stage, we randomly selected 60 primary sampling units using a list of city blocks published by the City Government of Wuhan. One hour before the start of the selected time interval, the interviewers went to the selected side of the city block and drew a map of the people working on the pavement, on the street, or inside the houses, huts or other constructions facing the street (see Figure 2 for an illustration). People who were moving through the street block and customers were not mapped. The people on the map were then enumerated. In order to achieve a self-weighting sampling scheme, in the second sampling stage an equal proportion (5%) of the mapped individuals in each primary sampling unit were randomly selected and asked for their consent to participate in the study [46].

We used estimates of the distribution of cluster sizes and the average intraclass correlation coefficient from the pilot to calculate the design effect due to clustering, using an approximation proposed by Holt [47]. We adjusted the sample size that would be needed to measure WTP with the desired precision (95% confidence interval (CI) with a margin of error of 5 RMB), if a simple random sample were drawn, in order to take into account clustering (using the estimated design effect) and refusals. The number of clusters (60) was chosen for operational reasons. The final sample size was 651.

### Questionnaire

The questionnaire had three parts. First, the interviewers described the purpose of the study and asked respondents whether they consented to participate. Second, the interviewers asked participants questions about a number of individual characteristics. Neither participants' names nor any other characteristics that would allow identification of the participants after the interview were elicited or recorded. Third, the interviewers described the basic health insurance to the study participants. The description included the BHI benefits (hospital, outpatient and emergency care), the deductible, coinsurance and reimbursement ceiling (including a picture depicting how different copayments affect coverage), as well as the payment vehicle for premium contributions (a monthly premium paid into a city-wide social risk pooling fund managed by the Wuhan Bureau for Social Security). The benefits as well as copayments were in line with the 1999 central government provisions about the BHI and decisions by the provincial government of Hubei Province [48,49] and were implemented in 1999 in at least one city in Hubei Province (Yichang) [50]. The deductible was 9% of the average annual salary of formal sector workers in the jurisdiction or 700 RMB; the coinsurance was 10% for inpatient services, 20% for special procedures such as diagnostic imaging, cancer chemotherapy and dialysis, and 30% for outpatient services; the reimbursement ceiling was 4 times the average annual salary of formal sector workers or 30,000 RMB. Respondents were asked about their maximum WTP for baseline BHI and, separately, its three variations (i.e. BHI without ceiling, without deductible, and without coinsurance).

The central government proposal specifies that the BHI should be administered at the city level. In addition to payments into a city-wide social risk pooling fund the BHI for formal sector workers also includes medical savings accounts (MSA). Money that accrues in the MSA is reserved to pay for health expenditure. Funds in the MSA that are unspent at the end of a person's life are inheritable [51]. We did not include MSA in the specification of the payment vehicle in the WTP question, because it would have made the valuation more difficult to understand and the WTP responses more difficult to interpret (as respondents may value MSAs not only as a form of health insurance but also as a form of life insurance). Further, it is unclear whether or not the BHI for informal sector workers should include MSA.

### Demand curve fitting

Fitting demand curves from WTP data requires the selection of a functional form. We used the following criteria in the selection. First, at a premium contribution of zero all respondents should be willing to obtain the BHI. Second, the demand for BHI should (asymptotically) reach zero. Third, all else equal a simpler functional form should be preferred over a more complicated one. Fourth, the functional form should fit the WTP data better than other functional forms that satisfy the first three criteria [52]. Experimentation revealed that the simple exponential model

*Q *= 100*e*^b*WTP^

where

*Q *= proportion of the sample willing to pay at least the WTP amount, *WTP*, satisfied all four criteria. We estimated the exponential model iteratively by least squares regression.

### Equity weighting

In order to evaluate the social benefit of the BHI for informal sector workers, the individual WTP values need to be aggregated. One way to aggregate individual WTP is to sum up the WTP values as they are stated by the respondents, i.e. give everybody's WTP equal weight. However, WTP is a function of ability to pay. Weighting everybody's WTP equally will only then lead to results that can be considered fair if either the current income distribution is fair or all individuals have identical, linear utility functions. It is unlikely that the current income distribution in China is considered fair. For instance, the Chinese government is currently implementing large anti-poverty programmes in rural and urban China [53]. There is little empirical support for linear utility functions [54].

In order to derive a weighting scheme that takes account of equity concerns in the best possible way (i.e. maximizes social welfare) one needs to assume specific functional forms for individuals' utility functions and the social welfare function to be used to aggregate individual utilities. The choice of the utility function is a matter of empirical evidence; the choice of the social welfare function involves a value judgment about society's view on equity. We assumed that individuals' utility functions are identical and isoelastic (i.e. income elasticity of marginal utility is constant)

ui=yi(1−e)(1−e)
 MathType@MTEF@5@5@+=feaafiart1ev1aaatCvAUfKttLearuWrP9MDH5MBPbIqV92AaeXatLxBI9gBaebbnrfifHhDYfgasaacH8akY=wiFfYdH8Gipec8Eeeu0xXdbba9frFj0=OqFfea0dXdd9vqai=hGuQ8kuc9pgc9s8qqaq=dirpe0xb9q8qiLsFr0=vr0=vr0dc8meaabaqaciaacaGaaeqabaqabeGadaaakeaacqWG1bqDdaWgaaWcbaGaemyAaKgabeaakiabg2da9maalaaabaGaemyEaK3aa0baaSqaaiabdMgaPbqaaiabcIcaOiabigdaXiabgkHiTiabdwgaLjabcMcaPaaaaOqaaiabcIcaOiabigdaXiabgkHiTiabdwgaLjabcMcaPaaaaaa@3D97@

where

*u*_*i *_= individual *i*'s utility

*y*_*i *_= income

*e *= income elasticity of marginal utility or 'inequality aversion parameter'

and that the social welfare function is utilitarian, i.e. every person's utility receives equal weight in the aggregation of utililities across individuals

SW=∑i=1nui
 MathType@MTEF@5@5@+=feaafiart1ev1aaatCvAUfKttLearuWrP9MDH5MBPbIqV92AaeXatLxBI9gBaebbnrfifHhDYfgasaacH8akY=wiFfYdH8Gipec8Eeeu0xXdbba9frFj0=OqFfea0dXdd9vqai=hGuQ8kuc9pgc9s8qqaq=dirpe0xb9q8qiLsFr0=vr0=vr0dc8meaabaqaciaacaGaaeqabaqabeGadaaakeaacqWGtbWucqWGxbWvcqGH9aqpdaaeWbqaaiabdwha1naaBaaaleaacqWGPbqAaeqaaaqaaiabdMgaPjabg2da9iabigdaXaqaaiabd6gaUbqdcqGHris5aaaa@39FF@

where

*SW *= social welfare.

It can be easily shown that social welfare will be maximized if individual WTP, *WTP*_*i*_, are aggregated according to the following weighting scheme

∑i=1n(y¯yi)eWTPi
 MathType@MTEF@5@5@+=feaafiart1ev1aaatCvAUfKttLearuWrP9MDH5MBPbIqV92AaeXatLxBI9gBaebbnrfifHhDYfgasaacH8akY=wiFfYdH8Gipec8Eeeu0xXdbba9frFj0=OqFfea0dXdd9vqai=hGuQ8kuc9pgc9s8qqaq=dirpe0xb9q8qiLsFr0=vr0=vr0dc8meaabaqaciaacaGaaeqabaqabeGadaaakeaadaaeWbqaamaabmaabaWaaSaaaeaacuWG5bqEgaqeaaqaaiabdMha5naaBaaaleaacqWGPbqAaeqaaaaaaOGaayjkaiaawMcaamaaCaaaleqabaGaemyzaugaaOGaem4vaCLaemivaqLaemiuaa1aaSbaaSqaaiabdMgaPbqabaaabaGaemyAaKMaeyypa0JaeGymaedabaGaemOBa4ganiabggHiLdaaaa@4073@

where

y¯
 MathType@MTEF@5@5@+=feaafiart1ev1aaatCvAUfKttLearuWrP9MDH5MBPbIqV92AaeXatLxBI9gBaebbnrfifHhDYfgasaacH8akY=wiFfYdH8Gipec8Eeeu0xXdbba9frFj0=OqFfea0dXdd9vqai=hGuQ8kuc9pgc9s8qqaq=dirpe0xb9q8qiLsFr0=vr0=vr0dc8meaabaqaciaacaGaaeqabaqabeGadaaakeaacuWG5bqEgaqeaaaa@2E3F@ = average income,

i.e. the WTPs of workers with below average income are given weights greater than one and the WTPs of workers with above average income are given weights less than one [54]. The larger is the inequality aversion parameter *e*, the more averse to income inequality is a society. Such an equity weighting has been proposed for use in applied cost-benefit analysis [55,56]. While there is no published study about the shape of the marginal utility of income function in China, there is empirical evidence from India [55,57] and developed countries [55,58-60] that the marginal utility of income is indeed constant. Most empirical studies have found values of *e *between 1.0 and 2.0 [58,61]. We thus used three different values of *e *in our calculations: 0.5, 1.5 and 2.0. Because the BHI pools health risks at the city level [6], we used the average income in Wuhan in 2001 as an estimate of y¯
 MathType@MTEF@5@5@+=feaafiart1ev1aaatCvAUfKttLearuWrP9MDH5MBPbIqV92AaeXatLxBI9gBaebbnrfifHhDYfgasaacH8akY=wiFfYdH8Gipec8Eeeu0xXdbba9frFj0=OqFfea0dXdd9vqai=hGuQ8kuc9pgc9s8qqaq=dirpe0xb9q8qiLsFr0=vr0=vr0dc8meaabaqaciaacaGaaeqabaqabeGadaaakeaacuWG5bqEgaqeaaaa@2E3F@.

### Independent variables

We collected information on individual characteristics that were hypothesized to be determinants or correlates of WTP for health insurance as independent variables in multiple regression analysis [39]. We expected WTP to increase with age (because morbidity increases with age), income (because, holding other factors constant, higher income should lead to higher demand of all goods that are not inferior goods and there is no evidence to suggest that health insurance is an inferior good), and education (because individuals who have a low time preference should be both more likely to invest in education and more willing to pay for insurance).

In addition, we asked participants about their migration status, employment status, and health expenditure in the past year. We hypothesized that migrants have a lower WTP for BHI than Wuhan residents, because they are more likely than residents to leave Wuhan in the future and the BHI reimburses health expenditure only if it accrues in Wuhan. We further expected that, after controlling for income, permanently employed workers have a higher WTP for health insurance than workers who are only temporarily employed. The BHI guarantees coverage only if all monthly contributions have been paid up to the point in time when health expenditure accrues. Net of other factors, workers who are in temporary employment should be more likely than workers in permanent employment to default on their payments for the BHI in the future and thus have lower WTP for BHI in the present. Furthermore, respondents who have incurred higher health expenditure in the recent past are likely less healthy than those with lower past health expenditure and should thus be expected to be willing to pay more for participation in the BHI. Finally, we included sex as an independent variable because other studies have found significantly higher or lower WTP for health insurance among women than among men [25,30,31,34] (possibly, because gender roles influence health care-seeking behaviour and health insurance decisions). We logarithmically transformed the income variable and the health expenditure variable in order to reduce skewness in their distributions. See Table 1 for the definitions, means and standard deviations of the independent variables.

**Table 1:** Independent variables  (N = 609)

**Variable name**	**Definition**	**Mean**	**SD**
AGE	Age in years	35.6	10.8
MALE	Dummy variable = 1 if sex is male, 0 if female	0.49	0.50
EDU2	Dummy variable = 1 if highest educational attainment above elementary school but not above middle school, 0 otherwise	0.35	0.48
EDU3	Dummy variable = 1 if highest educational attainment above middle school but not above high school, 0 otherwise	0.39	0.49
EDU4	Dummy variable = 1 if highest educational attainment above high school, 0 otherwise	0.11	0.31
INCOME	Monthly income (RMB)	874.8	760.2
MIGRANT	Dummy variable = 1 if migration status is migrant, 0 if resident	0.21	0.41
EMPLOY	Dummy variable = 1 if employment is permanent, 0 if employment is temporary	0.8	0.40
HEX	Average monthly health expenditure in the past year (RMB)	27.4	72.1

SD = standard deviation, RMB = Renminbi

### Statistical analyses

On a payment card, the line of all positive WTP amounts is split into *j *mutually exclusive and exhaustive categories. The true WTP, WTP*, lies between the highest WTP value circled on the card, *PC*_*j*_, and the next highest value, *PC*_*j*+1_, where *j *= *0*,..., *J*. When regressing such an interval-coded dependent variable against independent variables

WTP* = ***Xβ ***+ ***ε***

the probability that WTP* falls in the *j*th category is given by

*P*(*WTP*_i_* = *F*(*PC*_*j *_- ***x***_***i***_***β***/*σ*) - *F*(*PC*_*j*+1 _- ***x***_***i***_***β***/*σ*)

where *F*(·) is the standard normal cumulative distribution function evaluated at (·). The likelihood function is the product of these probabilities across the *j *categories and over the *n *observations

L=∏i=0n∏j=0J[F(PCj−xiβ/σ)−F(PCj+1−xiβ/σ)]δij
 MathType@MTEF@5@5@+=feaafiart1ev1aaatCvAUfKttLearuWrP9MDH5MBPbIqV92AaeXatLxBI9gBaebbnrfifHhDYfgasaacH8akY=wiFfYdH8Gipec8Eeeu0xXdbba9frFj0=OqFfea0dXdd9vqai=hGuQ8kuc9pgc9s8qqaq=dirpe0xb9q8qiLsFr0=vr0=vr0dc8meaabaqaciaacaGaaeqabaqabeGadaaakeaacqWGmbatcqGH9aqpdaqeWbqaamaarahabaWaamWaaeaacqWGgbGrcqGGOaakcqWGqbaucqWGdbWqdaWgaaWcbaGaemOAaOgabeaakiabgkHiTGqadiab=Hha4naaBaaaleaacqWFPbqAaeqaaGGadOGae4NSdiMaei4la8ccciGae03WdmNaeiykaKIaeyOeI0IaemOrayKaeiikaGIaemiuaaLaem4qam0aaSbaaSqaaiabdQgaQjabgUcaRiabigdaXaqabaGccqGHsislcqWF4baEdaWgaaWcbaGae8xAaKgabeaakiab+j7aIjabc+caViab9n8aZjabcMcaPaGaay5waiaaw2faamaaCaaaleqabaGae0hTdq2aaSbaaWqaaiabdMgaPjabdQgaQbqabaaaaaWcbaGaemOAaOMaeyypa0JaeGimaadabaGaemOsaOeaniabg+GivdaaleaacqWGPbqAcqGH9aqpcqaIWaamaeaacqWGUbGBa0Gaey4dIunaaaa@6359@

where *δ*_*ij *_= 1 if the *i*th observation falls in the *j*th category and 0 otherwise. Estimation of the model based on maximum likelihood, so-called interval regression, produces consistent and asymptotically efficient coefficient estimates [62].

Interval regression has been specifically recommended as the most appropriate regression method for analyzing results from contingent valuation studies using the payment card elicitation method [63] and has been used in many applied contingent valuation studies (e.g. [64,65]). As a robustness check we ran ordinary least squares regressions (OLS) using the midpoint of the reported WTP interval. Interval regression and OLS assume that the error term is normally distributed. Because the distribution of WTP is skewed, normality of the error terms was more closely approximated when WTP was logarithmically transformed. Hence we used the natural logarithm of WTP as our dependent variables (i.e. we logarithmically transformed the boundaries of the payment card intervals for interval regression and the midpoint of the payment card intervals for OLS regression). We estimated

ln*WTP*_*i *_= *β*_0 _+ *β*_1_* AGE*_*i *_+ *β*_2_* MALE*_*i *_+ *β*_3_* EDU*2_*i *_+ *β*_4_* EDU*3_*i *_+ *β*_5_* EDU*4_*i *_+ *β*_6_ ln*INCOME*_*i *_+ *β*_7_* MIGRANT*_*i *_+ *β*_8_* EMPLOY*_*i *_+ *β*_9_ ln*HEX*_*i *_+ *ε*_*i*_

We adjusted the standard errors of the means of the unweighted and equity weighted WTP values, as well as the standard errors of the regression coefficients for intraclass correlation due to the clustering of individuals in the primary sampling units [66]. All analyses were performed with Stata 9.0 (Stata Corporation, College Station, Texas, USA).

## Results

### Sample

Out of 651 people who were asked whether they would be willing to participate in the study 621 consented to participate. Among the 621 participants 65 (i.e. 10.5%) answered zero to all four WTP questions. 11 answers were classified as protest zeros because respondents stated that "the BHI will not cover what it promises to cover" or "government should provide free health care for all" or that they were "too busy to bother with such things". In addition to the 11 respondents who gave protest-zero responses, one respondent was dropped from further analyses because he did not respond to the WTP questions, leaving a final sample of 609 respondents for analysis. The exclusion of protest zeros, did not significantly change the mean WTP for any of the four BHIs. When respondents who gave either a protest-zero response or did not answer the WTP questions were counted as non-responders, the response rate was 93.5%.

79% of the 609 informal sector workers included in our final sample were residents in Wuhan, while 21% were migrants. 80% of the workers had permanent and 20% had temporary employment (Table 1). Among residents, 69% were self-employed, 27% were employees and 4% were employers in enterprises with less than 10 people engaged. Among migrants, 97% were self-employed and 3% were employed in an enterprise with less than 10 people engaged. At the time of the study, not one of the respondents had health insurance coverage.

### WTP for different types of BHI

The mean WTPs for baseline BHI and its three variations are displayed in Table 2. The first row shows the unweighted values. On average, respondents were willing to pay significantly more for any of the three variants of BHI than for the baseline BHI (all p < 0.0001).

**Table 2:** Willingness to pay for basic health insurance (N = 609)

	**WTP for baseline BHI**		**WTP for BHI without ceiling**		**WTP for BHI without deductible**		**WTP for BHI without coinsurance**	
	Mean	95% CI	Mean	95% CI	Mean	95% CI	Mean	95% CI

Non-equity weighted	30	27–33	51	46–56	43	37–49	47	40–54
Equity weighted								
*e *= 1.0	38	32–43	66	55–76	56	46–65	59	48–70
*e *= 1.5	67	47–86	121	83–159	102	70–134	107	69–145
*e *= 2.0	166	93–239	310	170–450	263	145–382	275	133–417

All WTP values in Renminbi.  WTP = willingness to pay, BHI = basic health insurance, CI = confidence interval

In comparison to the baseline BHI, respondents were willing to pay 70% more for BHI without ceiling, 43% more for the BHI without deductible and 56% more for the BHI without coinsurance. Among the 609 respondents who gave valid answers to the four WTP questions only 9, 20 and 27 were willing to pay more for the baseline WTP than for the BHI without ceiling, the BHI without deductible, and the BHI without coinsurance, respectively. Mean WTP ranked as follows: WTP for BHI without ceiling > WTP for BHI without coinsurance > WTP for BHI without deductible > WTP for baseline BHI. However, the means of the WTP for BHI without ceiling, without deductible, and without coinsurance were not significantly different from each other at the 5% confidence level.

Respondents were willing to pay substantial proportions of their income for the different BHIs: 4.6% for the baseline BHI (95% CI, 4.1–5.1%); 7.8% for BHI without ceiling (95% CI, 7.0–8.8%); 6.8% for BHI without deductible (95% CI, 5.9–7.6%); and 6.9% for BHI without coinsurance (95% CI, 6.2–7.8%). Table 3 shows the mean WTP (and the 95% CI) for the baseline BHI and its three variations by age group, sex, educational attainment, income group, migration status, employment status, and past health expenditure.

**Table 3:** Willingness to pay for basic health insurance by respondents' characteristics

	**WTP for baseline BHI**		**WTP for BHI without ceiling**		**WTP for BHI without deductible**		**WTP for BHI without coinsurance**	
	Mean	95% CI	Mean	95% CI	Mean	95% CI	Mean	95% CI

**Age group (years)**								
18–30	33	28–39	55	45–64	51	37–66	51	36–65
31–40	31	26–35	53	45–61	42	36–48	47	39–55
41–50	24	20–28	42	34–50	36	27–44	46	29–64
51–60	26	19–33	45	34–57	34	25–42	39	29–50
**Sex**								
Female	33	29–38	55	47–64	50	39–61	55	42–67
Male	26	23–29	46	41–51	37	33–41	40	35–45
**Educational attainment**								
Elementary school or below	20	15–24	32	25–39	25	20–31	29	22–35
Above elementary school but not above middle school	27	23–31	46	39–53	39	33–45	42	34–50
Above middle school but not above high school	34	29–39	57	48–66	52	38–65	56	41–71
Above high school	39	30–48	70	55–86	53	43–64	60	44–75
**Monthly income (RMB)**								
0–499	15	12–19	26	20–32	23	17–28	22	17–27
500–999	25	23–28	42	38–47	35	32–39	39	34–43
1000 and above	53	44–61	91	75–106	80	58–102	88	63–114
**Migration status**								
Resident	32	28–35	53	48–59	45	38–53	51	42–59
Migrant	23	19–27	41	33–48	35	29–41	34	28–41
**Employment status**								
Temporary employment	19	16–23	33	26–39	27	23–32	27	22–32
Permanent employment	32	29–36	55	49–61	47	40–54	52	44–61
**Average monthly health expenditure in the past year (RMB)**								
0–99	29	26–32	48	43–53	42	36–48	45	38–52
100 and above	45	34–57	92	70–115	64	49–80	83	58–108

WTP = willingness to pay, BHI = basic health insurance, CI = confidence interval, RMB = Renminbi

### Demand curves

**Figure 3:**
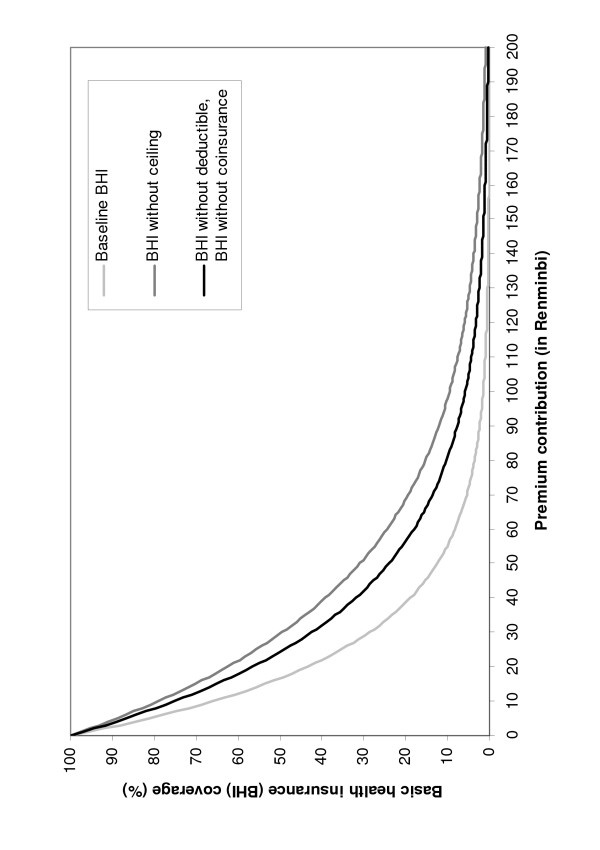
Demand for basic health insurance.

Table 4 presents the results of the nonlinear regressions of BHI coverage against WTP. The coefficients of determination indicate a near perfect fit. Figure 3 displays the estimated demand curves. Unlike the convention in economics, quantity (i.e. coverage) is displayed on the y-axis and price (i.e. WTP or maximum premium contribution) on the x-axis. At low levels of premium contribution, coverage declined rapidly with increasing contributions. For instance, 43% of the sample were willing to pay 20 RMB or more for the baseline BHI, but only 29% were willing to pay 30 RMB or more.

**Table 4:** Demand curve regressions

	***b***	**t ratio**	**p**	**Adj. *R***^2^	**Number of data points**	**Sample size**
Coverage with baseline BHI	-0.0416	-51.77	< 0.0001	0.9987	16	609
Coverage with BHI without ceiling	-0.0235	-57.65	< 0.0001	0.9988	20	609
Coverage with BHI without deductible	-0.0285	-62.95	< 0.0001	0.9991	18	609
Coverage with BHI without coinsurance	-0.0284	-44.57	< 0.0001	0.9979	20	609

BHI = basic health insurance, *b *= regression coefficient

### Equity weighted aggregation of individual WTP

The means of all equity weighted WTP were considerably higher than the means of the non-equity weighted WTP and increased with the inequality aversion parameter *e*. In comparison to the non-equity weighted WTPs, the mean WTPs were 1.3 times higher if *e *= 1.0; 2.3 to 2.4 times higher if *e *= 1.5; and 5.9 to 6.1 times higher if *e *= 2.0. The ranking of the point estimates of WTP for the different types of BHI was robust across all three equity weighting schemes (BHI without ceiling > BHI without coinsurance > BHI without deductible > baseline BHI) (Table 2). However, the only significant differences at the 5% confidence level between means of WTP for different types of BHI were found between WTP for baseline BHI and each of its three variations without equity weighting or with equity weighting and an inequality aversion parameter *e *of 1.0.

### Comparison of WTP to estimates of the costs of BHI

This study did not include a bottom-up calculation of the costs of the BHI. We estimated BHI costs with two types of information. First, we approximated costs with the average premium contribution of a formal sector worker to the BHI. The baseline BHI for formal sector employees is stipulated to be financed by 4.2% of employees' pre-tax wages and the resulting contributions are expected to cover the costs of BHI [6]. The central government policy decrees that the employer contributes 6% and the employee contributes 2% of the employee's wage to the BHI system [6]. However, only 4.2% of the wages are used to finance the BHI as described in the WTP question in this study. The remaining 3.8% are saved in MSA [67]. Participants in the BHI can use the money in the MSA to pay for health care which is not reimbursed by the social risk pooling fund because of the deductible, coinsurance or ceiling of the BHI [6]. The benefits of the MSA were not included in the valuations of the baseline BHI in this study. In 2001, average annual pre-tax income in Wuhan was 7091 RMB [68]. The average monthly cost of BHI was thus estimated to be 24.8 RMB.

Second, we used the respondents' past health expenditure to approximate the costs of the BHI. Average monthly health expenditure in the past year was 27.4 RMB (95% CI 21.6–33.1) (see Table 1). If we applied a deductible of 700 RMB, average monthly health expenditure accruing to the BHI would be reduced to 18.1 RMB (95% CI 12.2–23.9). If we applied a coinsurance of 10% in addition to the deductible, average monthly expenditure would be further reduced (to 16.3 RMB). The mean non-equity weighted WTP for baseline BHI and all three equity weighted means of WTP for baseline BHI were significantly larger than the above cost estimates.

### Determinants of willingness to pay

The explained variation of the multiple regression models was measured by McKelvey and Zavoina's *R*^2 ^in interval regressions and by the coefficient of determination, *R*^2^, in OLS regressions [69]. It ranged from 21% to 28%. All models were statistically significant (all p < 0.0001). In all regressions the following factors significantly influenced WTP: age (AGE; p values between 0.0002 and 0.0356), sex (MALE; p values between 0.0003 and 0.0538), income (INCOME; all p < 0.0001), past health expenditure (HEX; p values between < 0.0001 and 0.0004) and employment status (EMPLOY; p values between 0.0009 and 0.0324) (Table 5). Migration status was significant at the 5% confidence level in predicting WTP for baseline BHI, WTP for BHI without ceiling, and WTP for BHI without coinsurance (MIGRANT; p values between 0.0001 and 0.0475) and borderline significant in predicting WTP for BHI without deductible (both p < 0.0800). The educational attainment variables (EDU2-4) were neither individually nor jointly significant in predicting WTP except for highest educational attainment above high school (EDU4), which significantly increased WTP in the models labelled 2.1, 3.1, 3.2 and 4.1 in Table 5.

**Table 5:** Determinants of willingness to pay

Dependent variables	WTP for baseline BHI (ln)		WTP for BHI without ceiling (ln)		WTP for BHI without deductible (ln)		WTP for BHI without coinsurance (ln)	
			
Model	1.1	1.2	2.1	2.2	3.1	3.2	4.1	4.2
Regression	Interval regression	OLS	Interval regression	OLS	Interval regression	OLS	Interval regression	OLS
			
AGE	-0.013	-0.014	-0.014	-0.016	-0.014	-0.015	-0.010	-0.012
	(0.0039)	(0.0043)	(0.0044)	(0.0048)	(0.0037)	(0.0041)	(0.0049)	(0.0052)
	0.0010	0.0018	0.0013	0.0017	0.0002	0.0003	0.0356	0.0294
MALE	-0.203	-0.221	-0.154	-0.166	-0.198	-0.212	-0.212	-0.226
	(0.0558)	(0.0635)	(0.0756)	(0.0843)	(0.0643)	(0.0721)	(0.0670)	(0.0745)
	0.0003	0.0010	0.0416	0.0538	0.0020	0.0046	0.0016	0.0036
EDU2	0.140	0.148	0.156	0.154	0.219	0.227	0.151	0.150
	(0.0978)	(0.1097)	(0.1260)	(0.1409)	(0.1126)	(0.1260)	(0.1076)	(0.1187)
	0.1527	0.1825	0.2157	0.2786	0.0522	0.0764	0.1614	0.2099
EDU3	0.084	0.083	0.133	0.122	0.207	0.206	0.165	0.163
	(0.1182)	(0.1311)	(0.1424)	(0.1579)	(0.1424)	(0.1575)	(0.1264)	(0.1390)
	0.4761	0.5275	0.3515	0.4443	0.1461	0.1951	0.1913	0.2488
EDU4	0.222	0.230	0.333	0.334	0.347	0.356	0.305	0.302
	(0.1527)	(0.1682)	(0.1645)	(0.1827)	(0.1561)	(0.1734)	(0.1470)	(0.1622)
	0.1458	0.1768	0.0429	0.0724	0.0262	0.0447	0.0379	0.0676
INCOME (ln)	0.439	0.468	0.469	0.499	0.458	0.489	0.434	0.461
	(0.0504)	(0.0542)	(0.0588)	(0.0642)	(0.0574)	(0.0622)	(0.0566)	(0.0613)
	< 0.0001	< 0.0001	< 0.0001	< 0.0001	< 0.0001	< 0.0001	< 0.0001	< 0.0001
MIGRANT	-0.345	-0.373	-0.214	-0.224	-0.174	-0.187	-0.287	-0.303
	(0.0888)	(0.1025)	(0.0979)	(0.1108)	(0.0915)	(0.1050)	(0.1033)	(0.1157)
	0.0001	0.0006	0.0285	0.0475	0.0566	0.0800	0.0054	0.0113
EMPLOY	0.224	0.232	0.258	0.273	0.191	0.198	0.290	0.300
	(0.0835)	(0.0910)	(0.0930)	(0.1022)	(0.0826)	(0.0905)	(0.0872)	(0.0962)
	0.0073	0.0133	0.0055	0.0098	0.0209	0.0324	0.0009	0.0028
HEX (ln)	0.076	0.083	0.139	0.148	0.090	0.098	0.087	0.093
	(0.0198)	(0.0210)	(0.0220)	(0.0234)	(0.0203)	(0.0216)	(0.0236)	(0.0248)
	0.0001	0.0002	< 0.0001	< 0.0001	< 0.0001	< 0.0001	0.0002	0.0004
Constant	0.008	-0.266	0.204	-0.026	0.265	0.019	0.182	-0.035
	(0.3315)	(0.3617)	(0.3853)	(0.4270)	(0.3893)	(0.4239)	(0.4001)	(0.4358)
	0.9815	0.4642	0.5957	0.9508	0.4965	0.9643	0.6495	0.9363
			
*N*	609	609	609	609	609	609	609	609
F		22.7		23.1		21.7		19.11
Log likelihood	-1308		-1486		-1433		-1475	
*R*^2^		0.247		0.259		0.242		0.213
McKelvey and Zavoina's *R*^2^	0.266		0.277		0.257		0.224	
p value	< 0.0001	< 0.0001	< 0.0001	< 0.0001	< 0.0001	< 0.0001	< 0.0001	< 0.0001

The numbers in the cells are regression coefficient, heteroscedasticity-robust standard error adjusted for clustering of the data in 60 primary sampling units (in brackets) and p value.

WTP = willingness to pay, BHI = basic health insurance, OLS = ordinary least squares regression, ln = natural logarithm

Since we used the natural logarithm of the four WTP as dependent variables, the coefficients in Table 5 are either semi-elasticities (if the independent variable is in natural units) or elasticities (if the independent variable is logarithmically transformed). An increase of one year in age decreased WTP by between 1.0% and 1.6%. Net of the other factors included in the regression models, men had lower WTP than women (between 15.4% and 22.6% lower), migrants had lower WTP than residents (between 17.4% and 37.3% lower), and informal workers in permanent employment had higher WTP than workers in temporary employment (between 19.1% and 30.0% higher). A 1% increase in monthly income increased WTP by between 0.434% and 0.499% and 1% increase in health expenditure in the past year increased WTP by between 0.076% and 0.148% (Table 5).

The regression results were largely unaffected by the choice of regression model. The coefficient sizes and the significance levels in the OLS regression models were similar to those in the interval regression models. Only two coefficients differed by more than 10%. The age coefficients in the OLS regression models labelled 2.2 and 4.2 in Table 5 were respectively 14% and 20% lower than the age coefficients in the corresponding interval regressions (models 2.1 and 4.1 in Table 5).

## Discussion

### Validity of WTP

Most informal sector workers in Wuhan are currently not eligible to participate in the BHI (see below). If the City Government of Wuhan decides to open the BHI to a broad range of informal sector workers, this study will offer a rare opportunity to assess the criterion validity of WTP responses by comparing actual demand to demand predicted from a contingent valuation study. For now, we can only examine the construct validity of the WTP responses, i.e. investigate whether WTP relates to certain constructs as predicted by theory. Two commonly used tests of construct validity of WTP are the income elasticity test and the scope test [70]. With the rare exception of inferior goods, demand theory predicts that income elasticity of goods is positive and there is no evidence to suggest that health insurance is an inferior good. We found that the income elasticity of BHI is indeed positive after controlling for sex, age, migration status, employment status and health expenditure. Our results thus pass the income elasticity test.

The scope test assesses whether WTP increases as more of a good is supplied [71,72]. We found that mean WTP significantly increases and that the demand curve is shifted upward as the scope of the BHI increases, i.e. as either the reimbursement ceiling, the deductible or the coinsurance are removed from the baseline BHI. A few respondents were willing to pay less for one of the variants of BHI than for the baseline BHI. However, this does not imply that the answers of these respondents are invalid. While the removal of the ceiling, the deductible or the coinsurance never decreases the scope of the BHI, respondents may truly value the BHI variants less than the baseline BHI because they associate other changes in the BHI with the change in scope. For instance, a respondent may expect that the BHI without coinsurance will be less sustainable than the BHI with coinsurance and thus value the former less than the latter.

Weaker tests of construct validity of WTP responses include assessments of the relationship between respondents' characteristics other than income. All our independent variables influenced WTP for BHI as hypothesized, with two exceptions. First, WTP decreases significantly with age. We had hypothesized that WTP for health insurance would be a positive function of age because the risk of disease increases with age. However, it is plausible that age not only proxies for disease risk but is also associated with factors that are not controlled for in our regression analyses and are negatively associated with WTP for BHI. Younger informal sector workers may be more likely than older workers to be employed in occupations that carry a high risk of accidents, for instance in construction. Also, the age variable may capture cohort effects. For instance, older informal sector workers may believe that their children will finance their health care should they become ill, while younger workers do not.

Second, the relationship between education and WTP was not very strong. We had hypothesized that higher education leads to higher WTP for BHI, because people who discount the future at a lower rate both invest more in their education and are willing to pay more in order to insure against future health expenditure. However, it seems plausible that educational attainment in China has in the past not been determined by preferences but by factors such as place of birth, party affiliation or early educational performance. Overall, tests of construct validity suggest that the results of our contingent valuation study are valid.

### Policy relevance

Health policy in China is formulated centrally, but decentralization has given lower levels of government power to adapt policies from higher levels to local circumstances, leading to a situation where "governments at all levels are both policy-makers and policy-implementers" [9]. Discrepancies between central policy directives and local policy implementation have been observed for several health policies in China, such as disease control policies [73] and health care price setting [74].

The introduction of the BHI in Chinese cities is an example of the simultaneity of policy formulation and implementation at the local level. While the 1998 central government policy includes detailed directives about the attributes of the BHI (e.g. the proportions of a formal sector worker's salary that employer and employee need to contribute to the BHI) [6], some municipal governments have implemented modified versions of the BHI, for instance in Shanghai and Beijing [75,76]. Municipal governments have even more discretion in making decisions about expanding BHI to informal sector workers. The 1998 central government policy allows such an expansion, but does not require it. Recently, however, different levels of the Chinese government within and outside the health sector have called for improved social protection, including health insurance, for urban informal sector workers [20,77-79].

In 2004, the City Government of Wuhan issued the *Wuhan City regulation about basic health insurance for urban informal sector workers*. The regulation defines the eligibility criteria for participation in the BHI such that most informal workers are, in fact, barred from joining [80]. First, only those informal sector workers who have a permanent household registration (*hukou*) in Wuhan can join, which excludes migrant workers from participation. Second, men can only join if they have been enrolled in the state-run old-age pension scheme for at least 30 years and women can only join if they have been enrolled for at least 25 years. Since only formal sector workers are entitled to participate in the state-run old-age pension scheme, this second condition effectively prevents all informal sector workers from participating in the BHI who have never worked in the formal sector. Moreover, it excludes all men who were laid-off after less than 30 years of formal sector work and all women who were laid-off after less than 25 years from joining the BHI. Third, men younger than 16 or older than 60 years of age and women younger than 16 or older than 55 years of age are not eligible to participate in the BHI. The upper age limits prevent many of those few former formal sector workers who have been working long enough in the formal sector to meet the second eligibility criterion from participating in the BHI, because they are too old.

A few cities in China allow broader categories of informal sector workers to participate in the BHI if they contribute the same proportion of their incomes as formal sector workers or pay premiums similar to the average absolute premium paid by formal workers [77,81,82]. However, participation in these voluntary BHI schemes has been low [82]. Workers in the formal sector pay only a part of the total contribution directly from their salaries, while their employers pay the remainder. In contrast, informal sector workers usually have to pay the total contribution from their own incomes because they are either self-employed or employed in small-scale enterprises that do not contribute to their employees' insurance coverage [83].

This is the first study to investigate WTP to participate in social health insurance among informal sector workers in a large Chinese city. Many factors, such as political will and financial, managerial and technical resources, will influence municipal governments' decisions to offer informal sector workers participation in the BHI. Our study may support the decision making by providing evidence about the social desirability of the BHI, preferences for BHI attributes, and characteristics of informal sector workers that influence the valuation of BHI.

On average, informal workers are willing to pay substantial absolute amounts and substantial proportions of their incomes to obtain social health insurance coverage. We find that average WTP is significantly higher than estimates of average cost of BHI based on the premium contributions of formal sector workers and past health expenditure of informal sector workers. From a normative perspective, the provision of the BHI to informal sector workers should thus increase social welfare.

Whether the net benefits of the BHI will be positive or not depends on the validity of our cost estimates. For our first cost estimate – the average premium contribution of formal sector workers – to accurately represent the average cost of BHI, the contribution rates that are stipulated in the 1998 central government policy need to be sufficiently high, so that the total premium contributions to the BHI cover total costs. The fact that in their implementation of the BHI for formal sector workers some cities have chosen contribution rates that are higher than the rates stipulated by the central government suggests that this assumption may not hold true [67]. For instance, in Shanghai employers are required to contribute 10% of an employee's annual wage to the BHI, while employees contribute 2% [75]. Even if the stipulated rates did lead to contributions that are sufficient to cover the costs of BHI for formal sector workers, they might not lead to contributions sufficient to cover the costs of BHI for informal sector workers, for instance because the latter face higher risks of work-related injuries and diseases than the former [82].

For our second cost estimate – the informal sector workers' past health expenditure, to approximate well the cost of BHI – the demand for health care must not be affected by insurance. However, insurance is likely to change the demand for health care because it decreases the price of health care (as well as workers' disposable incomes), and may lead to increased moral hazard behaviours.

While our cost estimates may thus be too low, the estimates of the benefits of BHI that do not take into account aversion to income inequality may underestimate the true size of the benefits from BHI. The equity weighted mean WTPs for BHI are much higher than non-equity weighted mean WTPs. If we assume, for instance, that the correct inequality aversion parameter for China is 1.5, the benefits of the BHI will be more than twice as high as the unweighted benefit estimates, so that net social benefit will be positive, unless costs have been underestimated by more than a factor 2. One indication that Chinese society is indeed inequality averse is that Chinese policy makers have stipulated that formal sector workers should contribute an equal proportion of their incomes to the BHI, i.e. to receive BHI coverage workers with higher incomes are required to pay larger absolute amounts into the social risk pooling fund than workers with lower incomes.

From a behavioural perspective, our result that coverage declines steeply with increasing premium contributions at low contribution levels suggests that government subsidies would be an effective mechanism to increase coverage with BHI among informal sector workers. Our results further suggest that informal sector workers do not value the BHI as a mechanism to recover the relatively frequent but small financial losses associated with common illnesses but because it protects against the rare but large financial losses associated with catastrophic care. The informal sector workers in our sample state a WTP that is on average higher than their health expenditure in the past year. In fact, only 62 of the 609 respondents would have received any money from the BHI at the level of their past year's health care spending; all other workers spent less on health care than the deductible. Nevertheless most workers are willing to pay positive amounts for the BHI, suggesting that workers value the protection against rare diseases that are expensive to treat. This conclusion is strengthened by our finding that average WTP for BHI increases significantly when the ceiling on coverage is removed, even though not one of the informal sector workers in our sample had health expenditure exceeding the ceiling in the past year.

A recent study investigated WTP for private health insurance in four small cities in China's Shandong and Sichuan Provinces [35]. As described above, this study differs from ours in important aspects, including the target population, the type of insurance and the setting. Nevertheless, it is interesting that the study finds that respondents value insurance for catastrophic diseases (such as cancer and end-stage renal disease) and inpatient care more highly than insurance for outpatient care. This reinforces our finding that people in urban China value financial protection against catastrophic care expenditure more highly than recovery of costs of treating minor diseases.

Our results suggest three potential limitations of a voluntary BHI for informal sector workers. First, voluntary BHI for informal sector workers may suffer from some adverse selection. We find that WTP increases significantly with health expenditure in the past year. As past health expenditure proxies future health expenditure [84] at any given premium contribution those who take out the BHI would be expected to incur higher health expenditure than those who do not. However, while past health expenditure significantly increases WTP, the effect is not very large. A 10% increase in past health expenditure leads to 0.76% increase in WTP for baseline BHI and to a 1.39% increase in WTP for BHI without ceiling. In comparison, a 10% increase in income will lead to a 4.39% increase in WTP for baseline BHI and a 4.69% increase in WTP for BHI without ceiling.

Second, large proportions of informal sector workers will choose not to buy the BHI at relatively low contribution levels. At a price of 25 RMB per month (i.e. our estimate of average monthly costs of the BHI based on the contributions of formal sector workers) 35% of informal sector workers will buy the BHI. At a price of 20 RMB coverage of informal sector workers will increase to 43%.

In order to increase coverage, policy makers should consider changing the attributes of the BHI. According to our results, at any given premium contribution the coverage that will be achieved if informal sector workers were offered the BHI without ceiling, without deductible or without coinsurance will be higher than the coverage that can be achieved if they are offered the baseline BHI. While the average WTP for BHI without ceiling is not significantly higher than the average WTP for BHI without deductible or coinsurance, almost all respondents ranked the BHI without ceiling highest (by the WTP criterion) among the four different types of BHI offered. Including catastrophic care cover in the BHI would have the additional advantage of improving the effectiveness of the BHI in ensuring access to needed health care and averting poverty, while removing the deductible or the coinsurance may come with the disadvantage of increasing consumer moral hazard. Our findings suggest that even if the government decides neither to subsidise nor change the BHI, coverage will increase over time as the Chinese economy continues to grow (because WTP for BHI increases significantly with income).

Third, at any given premium the most vulnerable informal workers will be least likely to buy the BHI. Holding other factors equal, workers who do not buy the BHI will on average be older, poorer, and more likely to be migrants and without permanent employment than workers who do buy the BHI. In order to increase coverage amongst the most vulnerable workers, policy makers may consider a number of means, including targeted public marketing campaigns, specific subsidies (for instance for older workers) or changes to the BHI that specifically increase the attractiveness of the BHI for vulnerable workers (for instance, portability of the BHI may increase coverage amongst migrants).

## Conclusion

Our results suggest that Chinese municipal governments should allow informal sector workers to participate in the BHI. From a normative perspective, BHI for informal sector workers is likely to increase social welfare. The increases in social welfare are likely to be larger if Chinese society is averse to income inequality. Our results further suggest that informal sector workers do not value the BHI as a mechanism to recover the relatively frequent but small financial losses associated with common illnesses, but because it protects against the rare but large financial losses associated with catastrophic care.

From a behavioural perspective, at a price level equal to the average premium contribution of formal sector workers 35% of informal workers should be expected to enrol in the BHI. Because coverage declines steeply with increasing premium contributions at low contribution levels, subsidies could be an effective means to increase coverage. According to our results, modifications of the copayment structure of the BHI, such as eliminating the reimbursement ceiling, or changes in other attributes, such as including portability, could increase population coverage. Moreover, coverage should expand as the Chinese economy continues to grow. Finally, adverse selection will be unlikely to be a large problem if the BHI is offered to informal sector workers.

## Abbreviations

BHI Basic Health Insurance

GIS Government Insurance System

LIS Labour Insurance System

WTP willingness to pay

RMB Renminbi

## Competing interests

The author(s) declare that they have no competing interests.

## Authors' contributions

TB, XZ RS designed the study. TB and XZ carried out the pilot survey and the main survey. TB performed the statistical analysis and drafted the manuscript. YL, XZ and RS were involved in the interpretation of the data and revised the manuscript critically for important intellectual content. All authors read and approved the final manuscript.

## Pre-publication history

The pre-publication history for this paper can be accessed here:


